# Proteomics and Metabolomics: Two Emerging Areas for Legume Improvement

**DOI:** 10.3389/fpls.2015.01116

**Published:** 2015-12-24

**Authors:** Abirami Ramalingam, Himabindu Kudapa, Lekha T. Pazhamala, Wolfram Weckwerth, Rajeev K. Varshney

**Affiliations:** ^1^International Crops Research Institute for the Semi-Arid Tropics (ICRISAT)Hyderabad, India; ^2^Department of Ecogenomics and Systems Biology, University of ViennaVienna, Austria; ^3^School of Plant Biology and Institute of Agriculture, The University of Western AustraliaCrawley, WA, Australia

**Keywords:** abiotic and biotic stresses, developmental process, functional genomics, signaling pathways, stress tolerance

## Abstract

The crop legumes such as chickpea, common bean, cowpea, peanut, pigeonpea, soybean, etc. are important sources of nutrition and contribute to a significant amount of biological nitrogen fixation (>20 million tons of fixed nitrogen) in agriculture. However, the production of legumes is constrained due to abiotic and biotic stresses. It is therefore imperative to understand the molecular mechanisms of plant response to different stresses and identify key candidate genes regulating tolerance which can be deployed in breeding programs. The information obtained from transcriptomics has facilitated the identification of candidate genes for the given trait of interest and utilizing them in crop breeding programs to improve stress tolerance. However, the mechanisms of stress tolerance are complex due to the influence of multi-genes and post-transcriptional regulations. Furthermore, stress conditions greatly affect gene expression which in turn causes modifications in the composition of plant proteomes and metabolomes. Therefore, functional genomics involving various proteomics and metabolomics approaches have been obligatory for understanding plant stress tolerance. These approaches have also been found useful to unravel different pathways related to plant and seed development as well as symbiosis. Proteome and metabolome profiling using high-throughput based systems have been extensively applied in the model legume species, *Medicago truncatula* and *Lotus japonicus*, as well as in the model crop legume, soybean, to examine stress signaling pathways, cellular and developmental processes and nodule symbiosis. Moreover, the availability of protein reference maps as well as proteomics and metabolomics databases greatly support research and understanding of various biological processes in legumes. Protein-protein interaction techniques, particularly the yeast two-hybrid system have been advantageous for studying symbiosis and stress signaling in legumes. In this review, several studies on proteomics and metabolomics in model and crop legumes have been discussed. Additionally, applications of advanced proteomics and metabolomics approaches have also been included in this review for future applications in legume research. The integration of these “omics” approaches will greatly support the identification of accurate biomarkers in legume smart breeding programs.

## Introduction

The legume crops such as chickpea (*Cicer arietinum*), common bean (*Phaseolus vulgaris*), cowpea (*Vigna unguiculata*), faba bean (*Vicia faba*), lentil (*Lens culinaris*), lupin (*Lupinus luteus*), mungbean (*Vigna radiata*), pea (*Pisum sativum*), peanut (*Arachis hypogaea*), pigeonpea (*Cajanus cajan*), and soybean (*Glycine max*) have greatly contributed in providing nutrition, food security, and environmental sustainability (Graham and Vance, 2003; Varshney et al., 2013a). However, a majority of them are grown in marginal environments, subjected to abiotic (e.g., drought, heat, cold, salinity, waterlogging, heavy metal toxicities etc.) and biotic stresses (e.g., anthracnose, bean rust, bacterial blight, *Fusarium* wilt etc.), thus limiting their productivity (Dita et al., 2006; Varshney and Tuberosa, 2013; Rodziewicz et al., 2014). Moreover, these environmental conditions severely affect rhizobia-legume symbiosis, which contributes to ~45% of nitrogen required for agriculture (Karmakar et al., 2015). Therefore, for the development of superior varieties with enhanced stress tolerance, it is extremely important to understand stress response mechanisms in legumes which include changes in gene expression, and the consequent variations in the transcriptome, proteome and metabolome.

The availability of high throughput and cost effective next generation sequencing (NGS) platforms as well as high throughput genotyping technologies, have facilitated the generation of massive genomic data for model as well as crop legumes. These platforms have been vital in producing the genome sequence assemblies for the following legumes: adzuki bean (Kang et al., 2015; Yang et al., 2015), chickpea (Varshney et al., 2013b), common bean (Schmutz et al., 2014), Lotus (Sato et al., 2008), Medicago (Young et al., 2011), mung bean (Kang et al., 2014), pigeonpea (Varshney et al., 2012), and soybean (Schmutz et al., 2010). Furthermore, whole genome-resequencing data are also becoming readily available for mining superior alleles (Varshney et al., 2009; Lam et al., 2010). Similarly, transcriptomics/gene expression studies, using a range of platforms, have been valuable for identifying candidate genes associated with tolerance/resistance to different stresses as well as several agronomic traits (Kudapa et al., 2013; Campbell et al., 2014; Brasileiro et al., 2015).

Although QTLs/candidate genes/alleles have been used in breeding programmes, it has been observed that structural/expression variation identified at the genetic level are not always translated into the “predicted” phenotype. Additionally, mechanisms involved in stress tolerance can be complicated, e.g., the involvement of metabolites, multigenes and post-translational modifications (PTM) which cannot be investigated by genomics or transcriptomics approaches (Mazzucotelli et al., 2008; Weckwerth, 2011a). In this context, proteomics and metabolomics are promising approaches to enhance our understanding of functional molecules on specific aspects of multigene families and PTMs, instead of analyzing the genetic code (DNA) or transcript (RNA) abundance which may not correlate with their corresponding proteins (Weckwerth, 2011b; Hossain et al., 2012).

Proteomics, defined as the high-throughput study of proteins, has taken the lead in plant biological research and stress responses, especially due to the increasing number of plant genomes being sequenced and released (Pandey and Mann, 2000; Weckwerth, 2011b; Jorrín-Novo et al., 2015). In addition, advancements in mass spectrometry (MS), quantitative methods and bioinformatics approaches have allowed comprehensive identification, quantitation, validation and characterization for a wide range of proteins from specific organ/tissue/cells (Glinski and Weckwerth, 2006). The information obtained through these advanced approaches are valuable for deciphering protein structure and complex mechanisms such as enzymatic and regulatory functions of proteins coded by specific genes (Bachi and Bonaldi, 2008; Wienkoop et al., 2010a; Nanjo et al., 2011; Abdallah et al., 2012). Furthermore, proteomics approaches provide valuable information such as protein levels associated with stress tolerance, the modifications in proteomes under stress that link transcriptomics and metabolomics analyses as well as the role of expressed genes in the functionally translated regions of the genome linked to traits of interest (Kosová et al., 2011). Many proteomics based publications, especially related to plant development and other biological phenomenon such as symbiosis in legumes are available in the model legumes and *Arabidopsis thaliana*, as well as in some crop plants such as rice (*Oryza sativa*), wheat (*Triticum aestivum*), maize (*Zea mays*), soybean, tomato (*Solanum lycopersicum*) and tobacco (*Nicotiana tabacum*) (Jorrín-Novo et al., 2015).

In addition to proteomics, metabolomics is another important approach of functional genomics in which the identification and quantitation of metabolomes (collection of metabolites or small molecules) within a cell, tissue or organism produced through cellular metabolism, connects the cellular biochemical activity with the phenotype (Weckwerth, 2003). Major plant metabolomics approaches include metabolic fingerprinting, metabolite profiling and targeted analysis (Fiehn et al., 2000; Halket et al., 2005; Shulaev, 2006). Depending on the objective of study, different metabolomics approaches or a combination of approaches are applied. Furthermore, the use of MS, bioinformatics tools and softwares, allows metabolites to be measured quickly, simultaneously, in large numbers from a small amount of sample, which are spatially localized within the biological material (Bhalla et al., 2005; Patti et al., 2012). Since metabolites are closer to the phenotype, they reflect gene expressions and different regulatory processes more accurately and metabolomics is a powerful tool to study plant molecular phenotypes in response to stresses (Scherling et al., 2010; Arbona et al., 2013; Doerfler et al., 2013). For example, under abiotic stress conditions, the plant metabolism is affected due to factors such as inhibition of metabolic enzymes, shortage of substrate, extreme demand for specific compounds and a combination of these factors. Thus, the plant undergoes metabolic reprogramming to adapt to the predominant stress conditions through the production of anti-stress components such as compatible solutes, antioxidants and stress-responsive proteins (Wienkoop et al., 2008a; Obata and Fernie, 2012; Doerfler et al., 2014). In crop breeding programs, there has been great interest in using metabolites as selection biomarkers, since metabolites integrate the complex interaction between genotype and the environment (Fernie and Schauer, 2009).

With proteomics and metabolomics emerging as cutting edge functional biology disciplines for understanding plant adaptation mechanisms to stresses at cellular and developmental stages in different plant systems, there has been great interest in applying the knowledge to understand responses in different crop plants. These approaches, integrated with information obtained from genomics data, allow accurate identification of candidate genes and pathways involved in important agronomic traits that can be applied in crop breeding programs (Langridge and Fleury, 2011).

In this article different proteomics and metabolomics tools available and the use of these approaches in legumes especially to understand stress response mechanism, cellular and developmental processes and symbiosis, have been discussed. The assemblage of information and resources available for these aspects of legume proteomics and metabolomics will facilitate our understanding and utilization of these resources for legume crop improvement programs.

## Proteomics approaches and challenges for legume research

Proteomics approaches have been implemented according to the objective of the study and are based on certain criteria such as (1) descriptive proteomics for classification of proteins, (2) comparative proteomics for comparison of protein profiles (genotypes, cells, organs, developmental stages, stress response, etc., (3) PTMs that determine how proteins are modified, (4) Protein-protein interactions (PPI) for identifying protein complexes and interacting networks, (5) proteinomics for studying protein structure and functional groups, and (6) translational proteomics that involves transfer of methodology and knowledge for crop improvement (Jorrín-Novo et al., 2015). Data obtained through above mentioned approaches together with bioinformatics, provide significant information on biological processes and stress tolerance mechanisms that can be applied in crop breeding programs (Salekdeh and Komatsu, 2007; Nanjo et al., 2011; Hu et al., 2015).

Furthermore, the comprehensive analysis using differential proteomics in complex systems has demanded development of new technologies to study the cell proteome. Each approach has advantages and disadvantages from both the conceptual and the methodological perspectives. Several approaches are being used to study proteomics in plants and these include electrophoresis and/or chromatography combined with chemical or metabolic labeling and MS. In the following section, we have discussed the gel-based system which is significant for differential expression profiling for studying legume stress responses, and the advantages of the technologically more advanced gel-free, quantitative systems. The application of these approaches for protein profiling in model and crop legumes for understanding stress responses have been addressed with successful examples where proteomics approaches have been utilized for the advancement of crop legumes research. In the interest of channeling the usage of more advanced approaches for legume crop improvement research, the application of more advanced approaches applied in other plant systems, have also been addressed.

### Gel-based system for protein differential expression profiling

Gel-based systems have been widely applied for protein differential expression analysis which involves the use of two-dimensional gel electrophoresis (2D-GE) or two-dimensional difference gel electrophoresis (2D-DIGE) for protein separation coupled with MS applications for the identification and quantitation of proteins (Kosová et al., 2011; Subramanian and Smith, 2013). The 2D-GE method has an advantage of resolving and visualizing thousands of spots corresponding to different molecular forms of same proteins. However, it has low sensitivity and is not suited for protein quantitation (Marouga et al., 2005; Abdallah et al., 2012). It was found that the use of fluorescent dyes in place of Coomassie can considerably increase the sensitivity and compatibility for MS analyses (Vanderschuren et al., 2013). These factors have led to the usage of a more refined gel electrophoresis method, called the 2D-DIGE which is based on pre-labeling of protein mixtures with fluorescence. Thus, 2D-DIGE is a much more sensitive method, which allows protein detection at sub-picomolar levels for highly precise quantitative proteomic studies (Marouga et al., 2005). Few studies have been reported in legumes, which employed 2D-DIGE for monitoring early response to symbiosis and pathogenesis, such as in the root proteome in Medicago (Schenkluhn et al., 2010) and parasitic infections in pea (Castillejo et al., 2012). The advantages and disadvantages of 2D-GE and 2D-DIGE have been reviewed before (see Griffin and Aebersold, 2001; Marouga et al., 2005; Abdallah et al., 2012).

For identification of proteins separated by 2D-GE, MS based techniques were found to have high accuracy, resolution and sensitivity but the sample preparation seemed to be laborious (van Wijk, 2001; Subramanian and Smith, 2013). Earlier, the usage of techniques such as matrix-assisted laser desorption ionization time-of-flight (MALDI-TOF) MS and electrospray ionization (ESI) MS with tandem MS (MS/MS) overcame the slower and less sensitive methods (Griffin and Aebersold, 2001; Griffin et al., 2001; van Wijk, 2001). In particular, the application of tandem MS methods provided sequence information for more peptides accurately for the identification of proteins and PTMs (Pandey and Mann, 2000; Bachi and Bonaldi, 2008). The use of MALDI ionization with two TOF analysers, (MALDI TOF/TOF) and liquid chromatography tandem MS (LC-MS/MS) have been fairly new technologies for protein identification that enhanced the pipeline for *de novo* assembly. The availability of these tools, together with the genome sequences of more plants and accessibility of sequence databases, intensified the identification of protein spots in different plants (see Jorrín-Novo et al., 2015). The most commonly used pipeline for the identification of proteins involves comparing the MS/MS spectra to the reference databases (Romero-Rodríguez et al., 2014). Although SEQUEST, MASCOT and X!Tandem are the widely used database search programs for protein identification, MassMatrix is also suited for tandem MS data (Xu and Freitas, 2009; Senkler and Braun, 2012).

### Gel-free proteomics tools and approaches for quantitation

Shot-gun proteomics is a gel-free “bottom-up” strategy, in which complex peptide fractions produced after proteolytic digestion of proteins are analyzed by LC-MS/MS. The protein identification rates are enhanced using different peptide or protein fractionation strategies (Abdallah et al., 2012). This approach allows high throughput and comprehensive investigation, by providing an overview of the organelle or cell-type proteomes (Glinski and Weckwerth, 2006). For large-scale proteomics, a multidimensional chromatography technique called multidimensional protein identification technology (MudPIT) has been used to separate complex protein samples prior to MS analyses to increase the proteome coverage and dynamic range (Washburn et al., 2001; Bachi and Bonaldi, 2008).

MS based protein quantitation strategies may include untargeted quantitation, (database dependent or database independent protein identification) or targeted absolute quantitation, which are important for biomarker discovery and protein stoichiometries in protein complexes (Wienkoop et al., 2008b; Weckwerth, 2011b; Schmidt and Urlaub, 2012). Relative quantitation is an unbiased, large-scale screening strategy, which is useful to detect proteins involved in unknown regulatory processes, protein modifications and mechanisms in systems biology. MS-based methods for protein quantitation can be label (stable-isotope *in vivo* or *in vitro* label) or non-label based (see Bachi and Bonaldi, 2008; Abdallah et al., 2012). Label based proteomics for relative quantitation consists of chemical labeling and metabolic labeling. The iTRAQ (isobaric tags for relative and absolute quantitation), is a chemical labeling approach and has been applied to study salinity stress tolerance in the phloem sap proteome of *Cucumis sativus* (Fan et al., 2015), leaf proteome of *Vitis vinifera* to understand heat stress response (Liu et al., 2014) and the root proteome of rice to investigate aluminum stress response (Wang et al., 2014). The platforms available for relative quantitation include Mass Accuracy Precursor Alignment (MAPA), ProtMax, MASS WESTERN, and PROMEX (Hoehenwarter et al., 2008; Weckwerth et al., 2014). Recently, a rapid shot-gun LC-MS approach for relative quantitation called full-scan (FS) selective peptide extraction (Selpex) was successfully used for generating a reference library of targeted peptides from leaf tissue of Medicago (Castillejo et al., 2014).

Selective reaction monitoring (SRM) or multiple reaction monitoring (MRM), which involves the use of triple-quadrupole (QQQ) MS is a quantitative, targeted and label-free approach, that complements untargeted shotgun methods due to its reliable quantitation of low abundance proteins in complex mixtures (Lange et al., 2008; Wienkoop et al., 2010b; Schmidt and Urlaub, 2012). This approach has been useful to measure a predetermined set of proteins that could constitute cellular networks or candidate biomarkers across samples consistent and reproducibly (Wienkoop et al., 2010b; Picotti and Aebersold, 2012). In Medicago, the MRM approach was applied for the absolute quantitation of sucrose synthase isoforms and N-metabolism enzymes in symbiotic root nodules as well as in the analysis of nodule metabolism under drought stress (Wienkoop et al., 2008b; Larrainzar et al., 2009).

### Protein profiling in legumes

Proteomic studies have been carried out predominantly in Medicago to investigate stress tolerance, seed physiology, plant growth, development and symbiosis which has immense significance in agricultural research (Colditz and Braun, 2010; Jorrín-Novo et al., 2015). A majority of studies have been carried out to investigate abiotic stress responses, e.g., drought tolerance in shoots, leaves and roots using gel-based as well as non-gel based approaches (Table 1). For instance, 26 differentially expressed proteins were identified using 2D-GE and ESI-LC-MS/MS approaches from leaf samples subjected to drought stress (Aranjuelo et al., 2011). The study revealed the regulation process involved in the synthesis of amino acids pertaining to osmoregulation. Using non-gel based LC-MS/MS approaches, root nodule proteome and symbiotic nitrogen fixation was studied under drought stress in Medicago (Larrainzar et al., 2007, 2009). The former study (Larrainzar et al., 2007) not only identified a large number of nodule proteins, but also grouped the drought responsive proteins from plant and bacteroid which was helpful in understanding the mechanism in which each symbiotic member responded to drought stress. The later study (Larrainzar et al., 2009) was important for the relative quantification of root nodule proteins and absolute quantification of a key enzyme in sucrose metabolism to understand the regulation of nitrogen fixation under drought stress. In order to understand the roles of diverse LEA proteins in developing seeds of Medicago, differential profiling was effectively used to identify proteins that may have a role in desiccation tolerance and seed longevity (Chatelain et al., 2012). The study of arbuscular mycorrhizal symbiosis in Medicago roots identified differential accumulation of 96 membrane proteins which could have a role during symbiosis when compared between the mycorrhizal and non-mycorrhizal roots. The study has also identified proteins that stimulated changes in membrane trafficking (Abdallah et al., 2014).

**Table 1:** **Key studies on protein differential expression analysis in response to various stress in some model and crop legumes**.

**Legume**	**Cell/Tissue/ Organ**	**Stress**	**Method**	**Protein differential expression and identification**		**References**
				**Differentially expressed proteins**	**Function**	
Medicago	Shoots	Salt, drought	nanoESI-LC-MS/MS	–	Protein regulation, photosystem (PS11)	Staudinger et al., 2012
	Shoots	Cadmium	2D-GE, MALDI-TOF-MS	17	Photosynthesis, chaperones	Aloui et al., 2011
	Leaves	Drought	2D-GE, ESI-LC-MS/MS	26	Metabolism, energy, protein storage	Aranjuelo et al., 2011
	Nodules	Drought	LC-MS/MS	16	Nodule plant and bacteoid protein	Larrainzar et al., 2007
	Nodules	Drought	LC-MS/MS	–	Sucrose synthase, symbiotic nitrogen fixation	Larrainzar et al., 2009
Chickpea	Microsomal fraction from aerial tissue	Dehydration	2D-GE, MALDI-TOF-TOF	184	Photosynthesis, transport, metabolism	Jaiswal et al., 2014
	Leaves	Cold	2D-GE with MALDI-TOF/TOF and/or with LC-MS/MS	70	Defense, signal transduction, storage	Heidarvand and Maali-Amiri, 2013
	ECM	Dehydration	2D-GE, LC-ESI-MS/MS	81	Cellular function	Bhushan et al., 2011
	ECM	Dehydration	2D-GE, ESI-Q-TOF-MS/MS	134	Cell wall modification, signal transduction, metabolism, defense	Bhushan et al., 2007
	ECM	Dehydration	2D-GE, ESI-TOF-MS	147	Molecular chaperones, cell signaling	Pandey et al., 2008
Common bean	Leaves	Drought	2D-DIGE, LC-MS/MS	130	Metabolism, photosynthesis, protein synthesis, proteolysis, defense	Zadražnik et al., 2013
Green gram	Roots	Cadmium	2D-GE and MALDI-TOF MS	23	Nutrient metabolism	Muneer et al., 2014
Peanut	Mature seeds	Water deficit	LC MS/MS	93	Glycolysis, sucrose and starch, fatty acid metabolism	Kottapalli et al., 2013
	Leaves	Water deficit	2D-GE, MALDI-TOF-MS, Q-TOF-MS/MS	79	Photosynthesis, signal transduction, energy, metabolism	Kottapalli et al., 2009
Pea	Seeds	Osmotic	2D-GE and MALDI-TOF-MS	230	Glycolysis, signal transduction, detoxification	Brosowska-Arendt et al., 2014
	Roots	Drought	2D-GE, MALDI-TOF/TOF and LC-ESI-QTOF	18	Flavonoid and sulfur metabolism	Irar et al., 2014
	Roots	Salinity	2D-GE, ESI-Q-TOF MS/MS	35	Defense, stress related	Kav et al., 2004
Soybean	Roots	Cold, osmotic	2D-GE, LC/nanoESI-MS	59	Signal transduction, secondary metabolism, defense, energy, protein synthesis, development, translocation,storage	Swigonska and Weidner, 2013
	Seedlings	Flooding	2D-GE, nano-LC-MS/MS	168	Metabolism, transportation, localization, Isoflavone reductase	Khatoon et al., 2012
	Leaves	Salinity	2D-GE, MALDI-TOF-TOF-MS	91	Stress related, proteolysis, protein biosynthesis, photosynthesis	Ma et al., 2012
	Leaves, hypocotyls and roots	Drought	2D-GE, nanoLC-MS/MS	57	Stress related, defense	Mohammadi et al., 2012
	Roots	Flooding	2D-GE, MALDI-TOF- MS, nanoLC MS/MS	70	Transportation, localization, storage, metabolism, cell wall modification, programmed cell death	Salavati et al., 2012
	Developing seeds	High temperature, humidity	2D-GE, MALDI-TOF-MS	42	Signal transduction, protein biosynthesis, photosynthesis, protein folding, defense, metabolism, regulation, secondary metabolite biosynthesis	Wang et al., 2012a
	Leaves	Fungus	2D-GE, MALDI-TOF-TOF-MS	41	Defense, carbohydrate metabolism, energy	Wang et al., 2012b
	Plasma membrane	Osmotic	2D-GE, nano-LC-MS/MS	96	Transport	Nouri and Komatsu, 2010
	Endoplasmic reticulum	Flooding	2D-GE and BN-PAGE, nano-LC-MS/MS	~50	Heat shock proteins, chaperonins	Komatsu et al., 2011
	Microsomal proteins	Cadmium	2D-GE, nanoLC-MS/MS	13 and 11	Stress, protein biosynthesis	Ahsan et al., 2010a
	Leaves, stems and roots	High temperature	MALDI-TOF-MS, nanoLC-MS/MS and protein sequencing	54, 35, 61	Defense photosynthesis, secondary metabolism, protein biosynthesis	Ahsan et al., 2010b
	Roots	Waterlogging	2D-GE, MALDI-TOF- MS, ESI-MS/MS	24	Signal transduction, programmed cell death, homeostasis and metabolism	Alam et al., 2010
	Hypocotyls, roots	Salt	2D-GE ESI-Q/TOF-MS/MS	>20	LEA protein, protease inhibitor	Aghaei et al., 2009
	Seedlings	Aluminum	2D-GE, MALDI-TOF-MS	39	Defense, signal transduction, protein folding, transport	Zhen et al., 2007

In addition, there is a significant contribution of proteomics for studying abiotic stress in soybean at subcellular, organ and whole plant levels. The methodologies applied and the major discoveries in these studies have been reviewed in a number of articles (see Hossain et al., 2013; Hossain and Komatsu, 2014a,b). A few important examples of proteomics approaches used for analyzing soybean abiotic stress are described here. For instance, osmotic stress in the plasma membrane of soybean seedling was studied using both gel-based and nanoLC MS/MS approaches (Nouri and Komatsu, 2010). While the former technique identified four up-regulated and eight down-regulated protein spots, the latter approach, identified 11 up-regulated and 75 down-regulated proteins, of which 7 were identified in both the studies. In dissecting the mechanism of Cadmium (Cd) uptake and distribution in soybean, 2D-GE and LC-MS/MS approaches were employed on contrasting soybean cultivars to identify 13 and 11 differentially expressed proteins, respectively. In this study, highly up-regulated proteins associated with lignin biosynthesis indicated that xylem lignification may be preventing the translocation of Cd (Ahsan et al., 2010a). For deciphering the mechanism of heat tolerance in soybean seedlings, 2D-GE, MALDI-TOF-MS, LC-MS/MS and protein sequencing were applied in which 54, 35 and 61 proteins were differentially expressed respectively in leaves, stems and roots, respectively (Ahsan et al., 2010b). The study showed that heat shock proteins (HSPs) and antioxidant defense related proteins were induced and identified different proteins involved in tissue specific and common defense mechanisms.

In other legume crops, there are reports of significant discoveries being made for understanding abiotic stress responses through protein profiling. In the case of chickpea, drought is one of the most important abiotic stresses that severely affect its productivity, for which efforts have been made to dissect the genetic basis of tolerance (Thudi et al., 2014; Varshney et al., 2014). Toward understanding proteomic response to dehydration stress in chickpea, a few protein profiling studies have been made available (Table 1). For instance, the investigation of a dehydration-responsive microsomal proteome with 2D-GE and MALDI-TOF/TOF identified 184 proteins that showed significant differential expression (Jaiswal et al., 2014). This study was significant in identifying a novel component involved in dehydration signaling called CaSUN1. In a different study, using 2D-GE with MALDI-TOF/TOF and LC-MS/MS, proteomic changes were identified at early stage of cold stress in chickpea leaves (Heidarvand and Maali-Amiri, 2013). The analysis indicated that energy resources and primary metabolites respond by recreating a new homeostasis in preparation for long-term cold stress adaptation. In an earlier study, 81 dehydration responsive proteins were identified from profiling analysis of the chickpea organellar proteome in two contrasting genotypes, with 2D-GE and LC-ESI-MS/MS (Bhushan et al., 2011). The study proposed that cell wall restructuring and the control of reactive oxygen species were mainly responsible for better adaptability to the stress.

A few studies have also been conducted in pea through protein profiling for understanding abiotic stress response and are being described here. For example, 2D-GE, MALDI-TOF/TOF, and LC-ESI-QTOF was employed to dissect the signaling pathway leading to inhibition of biological nitrogen fixation under drought stress (Irar et al., 2014). The study successfully identified 18 nodule proteins regulated by both pea and rhizobium genomes under drought stress and unraveling the SNF regulation machinery in nodules. In a different study, changes in protein accumulation in germinating seeds under different osmotic conditions were monitored with 2D-GE and MALDI-TOF-MS (Brosowska-Arendt et al., 2014). This work showed that under optimal conditions, proteins associated to glycolysis, fatty acids synthesis and detoxification under osmotic stress considerably decreased, while proteins involved in signal transduction and protection were accumulated. Similarly, investigation of salinity stress response in pea with 2D-GE and ESI-Q-TOF MS/MS approach revealed significant differential expression of 35 proteins, of which 10 pathogenesis-related (PR) proteins was reported for the first time to be involved in salinity stress response, possibly involving a new signal transduction pathway (Kav et al., 2004).

### Protein reference maps in legumes

Protein reference maps have been developed for model and crop legumes, which identify as many proteins as possible in a particular tissue or cell cultures at a given point of time. This provides useful insights into important plant processes, such as stress tolerance, nutrient uptake and symbiotic association with rhizobia (Brechenmacher et al., 2009). These resources will allow further studies for efficient proteomics applications for the crop legumes, especially through the classification and characterization of proteins related to development and stress tolerance important for unambiguous candidate gene identifications. Reference maps have been established in Medicago for cell suspension cultures, roots, leaves, stems, flowers and pods (Watson et al., 2003). A protein reference map generated for cell suspension culture of Medicago consisted of 1367 proteins with 907 unique accessions, which could identify a complete tricarboxylic acid cycle, a nearly complete glycolytic pathway, a partial ubiquitin pathway, enzymes involved in secondary metabolism through functional annotations (Lei et al., 2005). Other specialized proteome maps generated include Medicago mitochondria (Dubinin et al., 2011); cell wall (Gokulakannan and Niehaus, 2010); embryonic cell cultures generated from single protoplasts (Imin et al., 2004) and roots (Mathesius et al., 2011).

Lotus proteome reference maps for nodules and roots consist of 780 and 790 protein spots identified with 2D-GE, with 45% of the corresponding unique gene accessions common in both the tissues (Dam et al., 2014). The study showed that PTMs were more prominent in nodules rather than in roots. In addition to this, higher levels of proteins such as pathogen-related 10, heat shock, and redox processes related were found in the nodules prior to nitrogen fixation and nodulin related proteins were prevalent in mature nitrogen fixing nodules. In Lotus proteome reference maps were also generated for pod and seed development (Nautrup-Pedersen et al., 2010). Similar reference maps were also developed in crop legumes such as soybean and peanut. In soybean, a comprehensive proteome reference map was generated with 5702 proteins identified for a single root hair cell (Brechenmacher et al., 2012). A previous reference map in root hair cells identified 1492 proteins (Brechenmacher et al., 2009). Other proteome maps generated in soybean includes root apex and differentiated root zone (Mathesius et al., 2011); seed filling (Hajduch et al., 2005), leaf (Xu et al., 2006), and response to pathogen invasion (Mithöfer et al., 2002). Similarly, leaf proteome reference map was developed in peanut (Katam et al., 2010). In chickpea, reference maps were generated for understanding the complexity of plant nuclear proteins (Pandey et al., 2006) and membrane proteins (Jaiswal et al., 2012). In the case of pea, reference maps have been developed for vegetative tissues (Schiltz et al., 2004) and mature seeds (Bourgeois et al., 2009).

Proteome reference maps generated through gel-based approaches consist of gel images in which selected “spots” were linked with protein identity information with arrows and numbers (Senkler and Braun, 2012). However, web-based resources with interactive features of the reference maps have also been made available. Examples of these resources include the “Seed Proteome,” the “Rice proteome database,” and the “Arabidopsis seed proteome.” Recently, for constructing proteome reference map, a software tool called “GelMap” was developed (Rode et al., 2011). This tool was used for generating a 3D GelMap of Arabidopsis complex 1 in which its unique proteins constituents has been defined (Peters et al., 2013).

### Legume databases for proteomics analyses

Various databases have been developed that store a large resource of plant proteins from the proteome reference maps of legumes and other plants, such as the plant proteomics database PROMEX (Hummel et al., 2007; Wienkoop et al., 2012). A continuously growing database, PROMEX consists of 116,364 tryptic peptide product ion spectra entries of 48,218 different peptide sequence entries from Lotus, Medicago, common bean and soybean as well as other plants such as Arabidopsis, rice, etc. The database could be searched for whole experiments with an experimental ID, meta-information and single proteins and their corresponding peptide reference spectra. Furthermore, new LC-MS/MS analyses can be searched against this spectral library (http://promex.pph.univie.ac.at/promex/). LegProt (http://bioinfo.noble.org/manuscript-support/legumedb) is a legume specific protein database consisting of amino acid sequences translated from predicted gene models and 6-frame translations of tentative consensus sequences expressed sequence tags (ESTs) (Lei et al., 2011). The ProteomeXchange contains the dataset of seed phosphoproteins from Lotus (PXD000053, http://proteomecentral.proteomexchange.org/cgi/GetDataset?ID=PXD00053) and has been valuable for understanding the regulatory mechanisms of seed germination in legumes (Ino et al., 2014). This dataset contains a total of 721 phosphopeptides from 343 phosphoproteins in cotyledons and 931 phosphopeptides from 473 phosphoproteins in hypocotyls.

The Medicago PhosphoProtein Database (MPPD, http://phospho.Medicago.wisc.edu), contains 3457 unique phosphopeptides with 3404 non-redundant sites of phosphorylation on 829 proteins. This database represents the most comprehensive Medicago phosphorylation data, which allows browsing of identified proteins, searching proteins of interests, in addition to conducting BLAST searches of the database using peptide sequences and phosphorylation motifs as queries (Rose et al., 2012a). The Soybean Proteome Database (SPD, http://proteome.dc.affrc.go.jp/Soybean/) consists of a repository of functional analysis of abiotic stresses (flooding, drought, and salt). In total, it consists of 23 reference maps of soybean and proteins collected from several organs, tissues, and organelles of soybean (Ohyanagi et al., 2012). Recently, another database for storage, allergen, and anti-nutritional proteins from cultivated soybean called Soybean Protein Database (SoyProDB; http://bioinformatics.towson.edu/Soybean_Seed_Proteins_2D_Gel_DB/Home.aspx) has also been developed (Tavakolan et al., 2013). In the case of yellow lupin (*Lupinus luteus* L.), a seed-protein catalog has been developed. In this study, 736 proteins corresponding to 152 unique proteins have been deposited in the WORLD-2DPAGE repository (http://world-2dpage.expasy.org/repository/0066/; Ogura et al., 2014).

### Post-translational modifications (PTMs) in legumes

PTMs are required for the functionality of proteins that regulate processes and the subcellular localization that could be analyzed using proteomics approaches (Pandey and Mann, 2000; Seo and Lee, 2004; Downes and Vierstra, 2005). Although mostly identified through MS approaches, PTM analyses are not straightforward as protein identifications, the reason being peptide analyses do not show the expected molecular mass and therefore more protein samples are required (Pandey and Mann, 2000). However, advancement in phosphoproteomics procedures, e.g., the enrichment of phosphopeptides using immobilized metal affinity chromatography (IMAC) or proteins with aluminum hydroxide or titanium dioxide called metal oxide affinity chromatography (MOAC) (Wolschin et al., 2005) have improved the identification efficiency (Ndassa et al., 2006; Chen et al., 2010; Hoehenwarter et al., 2013; Beckers et al., 2014). Among the PTMs, phosphorylation is the major post-translational regulatory processes in all eukaryotes followed by ubiquitin and SUMO (Small Ubiquitin-like MOdifier) conjugations (Mazzucotelli et al., 2008).

The root proteome of the Medicago genotype, Jemalong A173457, revealed unique phosphopeptides that covers 3404 non-redundant sites of *in vivo* phosphorylation on 829 proteins (Grimsrud et al., 2010). The large scale phosphoproteomic study identified multiple sites of phosphorylation on a number of crucial proteins in rhizobial symbiosis initiation such as SICKLE, NUCLEOPORIN133 and INTERACTING PROTEIN OF DM13. Further, the rapid Nod-factors (NF) induced changes in the phosphorylation levels of 13,506 phosphosites in 7739 proteins was recently measured for rhizobia-legume symbiosis in Medicago which was found useful for quantifying phosphorylation, specifically associated with NF-signaling (Rose et al., 2012b).

In chickpea, a differential phosphoproteomic study in response to dehydration stress identified 91 phosphoproteins that are likely to be involved in cell defense, photosynthesis, photorespiration, molecular chaperones and ion transport. The study also identified multiple sites of phosphorylation in key regulatory and functional proteins (Subba et al., 2013). Recently, a nucleus-specific phosphoproteome map of 107 identified phosphoproteins was constructed in chickpea, which identified a collection of phosphoproteins involved in many cellular functions such as protein folding, signaling, gene regulation, DNA replication/repair/modification, metabolism, etc. (Kumar et al., 2014). In an earlier study, the PTMs of αAI (α-amylase inhibitor) were compared among transgenic pea and chickpea expressing αAI from common bean, with the processed form of the protein from several bean varieties. The αAI proteins displayed microheterogeneity due to differences in glycan addition frequency, variation in glycan processing and differences in C-terminal exopeptidase activity (Campbell et al., 2011). Thus, PTMs in common beans were also investigated on three seed defensive proteins αAI-1, αAI-2, and arcelin-5 (Young et al., 1999). The data showed that the proteolytic cleavage is required for the activation of the proteins, which resulted in the loss of the terminal Asn residue in αAI-1, and a minimum of seven residues from the C-termini of all three proteins. Additionally, a significant difference in the glycosylation patterns of αAI-1 and αAI-2 has been reported, although the proteins showed high sequence homology. Similarly in pea, the presence of multiple trypsin inhibitors (TI) isoforms were attributed to PTMs and particularly post-translational processing at the C-terminus during the desiccation stage of seed development resulted in the appearance of TI isoforms in pea (Domoney et al., 1995).

Histone modifications and histone variants are known to be vital for various biological processes. In this context, variants of soybean histone, H3 and H4 and their PTMs were reported, which revealed several distinct variants of soybean histone and their modifications that were different from Arabidopsis (Wu et al., 2009). The study thus, provides important biological information toward understanding histone modifications and their functional relevance in legumes.

This area of research possesses great promise for legume improvement as it is supported by advanced proteomics technologies, in particular, developments in the strategies for detection and selective isolation of proteins with known function. As discussed above functional properties of proteins are often regulated by PTMs of proteins and the numerous techniques developed can be applied to the global identification of PTMs and their processing sites in legumes.

### PPI approaches and application in legume research

PPIs provide functional knowledge about proteins by analysing the interacting partners and interactomics, which is the large-scale study of PPI networks using high throughput methods. This provides valuable insights for understanding cellular function, metabolism and signaling mechanisms (Braun et al., 2013; Stasi et al., 2015). Interactomes (maps of PPI) are constructed on the basis of experimental data and computational prediction of interactions (Stasi et al., 2015). Approaches used in PPI analyses and mapping can be genetic, biochemical or proteomics-based. The strategies involve direct interactions with binary methods such as yeast two-hybrid (Y2H) or split ubiquitin, analyses of protein complexes with co-immunoprecipitation or affinity purification followed by MS (AP-MS), imaging and database analyses (Pandey and Mann, 2000; Fukao, 2012; Braun et al., 2013; Stasi et al., 2015). The identification of minor PPIs are also essential for studying transient interactions and proteins of low-abundance, which may be functionally relevant (Fukao, 2012). In recent years, many proteins have been identified with advances in MS technologies and large-scale proteomics, however, the consequence of this is the increased number of false positive protein identifications. Therefore, for the accurate appraisal of PPI identification, the inclusion of independent experimental validation is required (Fukao, 2012). Y2H system is sensitive in detecting transient and unstable interactions, and is suitable for PPI mapping and generating high-throughput data with a fine resolution to understand cellular process at the systemic level. However, it has limitations with incidences of false-positives and false-negative interactions. The PPIs are usually validated with techniques such as pull-down assays, co-immunoprecipitation, *in-situ* hybridization (von Mering et al., 2002; Parrish et al., 2006; Brückner et al., 2009).

Although limited PPI based studies have been undertaken in legumes, the studies involving stress signaling networks, nodule formation and symbiosis have provided valuable information in understanding these processes. The Y2H system has been mostly used in model legumes to study symbiosis and nodulation mechanisms and in the crop legumes mainly to study stress responses (Table 2). For example in pea, using the Y2H system and validation using *in planta* co-immunoprecipitation, the PPI observed among the heterotrimeric G proteins and GPCR protein were shown to be relevant in the salt and heat stress signaling pathways (Misra et al., 2007). In another study, the exposure to salt and cold simultaneously stimulated the expression of genes encoding a protein kinase (*PsCIPK*) and a calcineurin B-like protein (*PsCBL*) in pea (Mahajan et al., 2006). Although immunofluorescence and confocal microscopy showed that the PsCBL was localized in the cytosol and PsCIPK in the outer membrane, Y2H analysis indicated that both the protein products interacted and this was supported with Western blots. In chickpea, a few candidate interactors were identified for the 1R-MYB for drought tolerance with the Y2H system (Ramalingam et al., 2015).

**Table 2:** **PPI analyses in some model and crop legume using Y2H and validated with other approaches**.

**Legume**	**Stress/Condition**	**Interacting components**	**Method of confirmation**	**References**
Lotus	Nodule development	LjNSP2 homodimers	β-galactosidase assay	Murakami et al., 2013
	Symbiotic signaling	SINA4 and SYMRK	BiFC	Den Herder et al., 2012
	Nodule development	CASTOR homodimer, POLLUX homodimer	BiFC	Charpentier et al., 2008
		SIP1 and SymRK	Pull down assay	Zhu et al., 2008
	Cell growth and differentiation	LjRac and LjRacGAP1	Affinity chromatography	Borg et al., 1999
Medicago	Nodulation signaling	RAM1 and NSP2	BiFC	Gobbato et al., 2012
		NSP1 and NSP2	BiFC	Hirsch et al., 2009
		DMI3 and IPD3	BiFC	Messinese et al., 2007
Chickpea	Salt	CaCIPK6 and NtCBL3	-	Tripathi et al., 2009
Cowpea	Osmotic and heat	VuDRIP and VuDREB2A	Antibiotic and X-α-Gal	Sadhukhan et al., 2014
Mungbean	Osmotic stress	VrUBC1 and AtVBP1	BiFC	Chung et al., 2013
Pea	Salt and heat	Gα subunit with the G_β_ subunit and phospholipase C at the calcium-binding domain	Co-immunoprecipitation	Misra et al., 2007
	Salt and cold	PsCIPK and PsCBL	β-galactosidase assay, Far-western blotting	Mahajan et al., 2006
Soybean	Cold	SCOF-1 and SGBF-1	β-galactosidase assay	Kim et al., 2001
	ABA, drought, cold, salt	GmMYB76 homodimers, GmMYB76 and GmMYB177, GmZIP46 homodimer, GmZIP46 and GmZIP62, GmZIP46 and GmMYB76.	β-galactosidase assay	Liao et al., 2008a
			β-galactosidase assay	Liao et al., 2008b
	Salt and heat	GmGBP1 with R2R3 domain of GmGAMYB1 in	X-Gal assay	Zhang et al., 2013

Bimolecular fluorescence complementation (BiFC) uses the yellow fluorescent protein (YFP), split into two-non-overlapping N-terminal (YN) and C-terminal (YC) fragments, where each fragment is cloned in-frame to the gene of interest, allowing the expression of fusion proteins. PPI detected *in planta* causes the detection of yellow fluorescence, which is not found for non-interacting pairs or non-fused YN/YC (Bracha-Drori et al., 2004). Although identification of PPI by screening a cDNA library is usually performed in yeast, BiFC technology was applied to screen an Arabidopsis cDNA library against a bait protein *in planta* since subcellular compartmentation and protein modifications differ between plant and yeast cells (Lee et al., 2012).

A challenging area in crops research would be the development of a proteome-wide PPI maps to understand the complex biological pathways and cellular networks. A high-throughput, Y2H system (HTP-YTH), suitable for mapping PPIs was described by Fang et al. (2002) to screen PPI in plants. This system involves a yeast gap-repair cloning and a selectivity that reduces false positives and negative clones with automation in laboratory procedures. This system has been used to study the defense signal transduction pathway in rice, where more than 100 genes were selected as “baits” for HTP-YTH screening in which many known and novel PPIs were identified. In Arabidopsis, the first binary PPI map for the interactome network of plants was developed (Arabidopsis Interactome Mapping Consortium, 2011). The construction of these maps in legumes should facilitate system biology approaches and will greatly benefit legume crop improvement programs.

## Metabolomics approaches in legumes

Plants synthesize specialized metabolites that define the biochemical phenotype of a cell or tissue and can be viewed as the end products of gene expression (Sumner et al., 2003). Quantitative and qualitative measurements of cellular metabolites provide a broad view of the biochemical status of an organism that could be used to monitor and assess gene function (Fiehn et al., 2000). Furthermore, metabolomics contributes significantly to the study of stress biology by identifying different compounds such as by-products of stress metabolism, stress signal transduction molecules, molecules that are part of plant acclimation process etc. (Weckwerth, 2003; Larrainzar et al., 2009). The identified metabolic compounds could be further studied by direct measurement or correlating with the changes in transcriptome and proteome expression and confirmed by mutant analysis. In this section, recent applications of metabolomics approaches in the area of legume development, symbiosis and stress response are discussed.

Metabolomics is a rapidly developing technology and at present metabolic fingerprinting and metabolite profiling approaches are being used. For wide coverage of the vast range of metabolites present, several analytical techniques involving separation and detection are implemented (Scherling et al., 2010; Doerfler et al., 2013, 2014). The separation technique is selective for certain groups of metabolites which includes gas chromatography (GC) for volatile and primary metabolites such as sugars and amino acids (Weckwerth, 2011a), LC for mainly secondary metabolites (Scherling et al., 2010; Weckwerth, 2011a), capillary electrophoresis (CE) for ionic metabolites to be separated (Soga et al., 2003; Soga, 2007) and ultra-performance liquid chromatography (UPLC). UPLC is a powerful technique which has high resolution, sensitivity and throughput than conventional high performance liquid chromatography (HPLC). The MS analysers have been commonly used for metabolite profiling, particularly those that provide accurate mass measurements such as FTICR_MS, Orbitrap-MS or TOF-MS due to its fast scan times with improved deconvolution, run times and high mass accuracy (Scherling et al., 2010; Weckwerth, 2010, 2011a; Obata and Fernie, 2012; Arbona et al., 2013; Doerfler et al., 2013, 2014). GC-MS has been widely used in plant metabolomics research and electron impact (EI) supports strong interfacing of GC with MS which allows fragmentation patterns to be highly reproducible. Major metabolomics approaches and their applications in legumes are described below and are summarized in Table 3.

**Table 3:** **Application of metabolomics approaches in stress, development and symbiotic processes in some legumes**.

**Legume**	**Stress/Condition, Tissue**	**Methods**	**Metabolites**	**References**
Lotus	Drought, shoots	GC-EI-TOF-MS	Serine, proline, arabitol	Sanchez et al., 2012
	Salt, shoots	GC-MS	Citric acid, malic acid	Sanchez et al., 2011
	Biodiversity and plant/plant competition, leaves	GC-TOF-MS and LC-Orbitrap-MS	Especially secondary metabolites, Flavonoids	Scherling et al., 2010
Medicago	Metabolic reprogramming, roots	GC-MS, LC-MS	Flavonoids, triterpenoid, starch hydrolysis metabolites	Watson et al., 2015
	Symbiosis, roots	MSI(MALDI)-MS imaging	Organic acids, amino acids, sugars.	Ye et al., 2013
		LC-ESI-TOF-MS	Oxylipins	Zhang et al., 2012
	Flavonoid profiling, roots	LC-MS	Flavonoids	Staszków et al., 2011
	Arbuscular mycorhizal symbiosis, roots	GC-MS, HPLC, LC-MS	Amino acids, fatty acids, isoflavonoids	Schliemann et al., 2008
	Signaling pathway roots	HPLC coupled to UV photodiode array and ESI ion-trap MS(HPLC-PDA-ESI-ITMS)	Phenylpropanoid, isoflavonoid	Farag et al., 2007
	Biotic, abiotic signaling pathway, roots	GC-MS, LC-MS	Primary metabolites, amino acids, organic acids, carbohydrate, shikimic acids, saponins	Broeckling et al., 2005
	Microbial, roots	Reversed-phase HPLC-PDA-ESI-MS (HPLC-PDA-ESI-MS)	Saponins	Huhman and Sumner, 2002
Pea	Drought, leaves	NMR	Amino acids	Charlton et al., 2008
Soybean	Aphid infection, leaves	CE-TOF-MS	Flavonoids, alkaloids, amino acids	Sato et al., 2013
	Defense, cotyledon	LC-MS, NMR	Prenylated-isoflavones	Cheng et al., 2011
	Flooding, roots, hypocotyls	CE-MS	Succinate, citrate, pyruvate	Komatsu et al., 2011
	Symbiosis, root hairs	GC-MS, UPLC-QTOF-MS	Isoflavonoids, fatty acids,carboxylic acids	Brechenmacher et al., 2010
	Metabolic profiling, leaves	GC-MS	Sugars, organic acids, fatty acids	Benkeblia et al., 2007
	GM and isogenic	CE–TOF-MS	Amino acids	García-Villalba et al., 2008
	Salt stress, seeds	HPLC-UV-ESI-MS	Isoflavonoids, saponins	Wu et al., 2008

### Metabolite profiling

Metabolite profiling is the simultaneous measurement of all or a set of metabolites in a given sample. Several analytical techniques such as nuclear magnetic resonance (NMR), GC-MS, LC-MS, capillary electrophoresis–MS (CE-MS) and Fourier transform infrared (FT-IR) spectroscopy have been reported for analysing the data from metabolite profiling (Sumner et al., 2003; Weckwerth, 2003; Shulaev, 2006). The advantages and disadvantages of each technique for metabolite profiling have been discussed previously (Fiehn et al., 2000; Roessner et al., 2000; Sumner et al., 2003; Weckwerth, 2003; Weckwerth et al., 2004a,b; Shulaev, 2006). The use of metabolic profiling has been limited in crop legumes but this approach has been successfully demonstrated in model legumes. For example, in Medicago, untargeted quantitative MS approach was used to profile metabolites treated with rhizobial Nod factors to study the metabolic changes between the symbionts (Zhang et al., 2013). The study showed decrease in concentration of (9)-HODE class of oxylipins upon Nod factor treatment *in planta* and together with jasmonic acid inhibited Nod factor signaling. This suggests an important role for the oxylipin pathway in Nod factor signaling in symbiosis. In a different study, under early salt and drought stress conditions, the involvement of certain metabolites in nutritional priming through symbiotic interaction of nodulated plants and N-fertilized Medicago has been reported (Staudinger et al., 2012).

Similarly, to understand drought acclimation in model and forage legumes, a comprehensive and progressive reprogramming of metabolic pathways were suggested for increased water stress in Lotus (Sanchez et al., 2012). Using GC coupled to electron impact ionization (EI)-TOF-MS (GC-EI-TOF-MS), this study reported gradual increase in most of the soluble molecules profiled. In addition, comparative metabolomics between the Lotus species showed the presence of metabolites that were conserved and unique in response to drought stress. Metabolite profiling using a combination of ionomic and GC-MS was conducted for the shoots of extremophile Lotus species, adapted to highly saline coastal regions and was compared with that of cultivated glycophytic grassland forage Lotus species, to understand salt tolerance mechanisms. The extremophile Lotus species was identified to have higher salt levels with a differential rearrangement of shoot nutrient levels upon salt exposure (Sanchez et al., 2011). In a similar study, the accumulation of alanine under anoxic conditions was examined in Lotus, which is highly tolerant to water logging (Rocha et al., 2010). High accumulation of succinate, alanine and the direct co-substrates for alanine synthesis, glutamate and gamma aminobutyric acid (GABA) in the roots of Lotus during water logging was reported. Whereas, majority of amino acids that are derived from TCA cycle intermediate were found to be decreased, which support earlier findings that the metabolic equilibriums are expected to drive the metabolic flux from glycolysis, via alanine synthesis and oxoglutarate to succinate, which prevents the accumulation of pyruvate activating fermentation leading to ATP production by succinyl- CoA ligase. In another study in Lotus, GC-TOF-MS (primary metabolism) and LC-Fourier-Transformation-MS (secondary metabolism) was applied to study plant/plant competition responses in a large biodiversity experiment (Scherling et al., 2010). Significant effects in Lotus were not associated with primary metabolism (sugars, amino acids, organic acids) but with the secondary metabolism. A significant gradient of several putative flavonoids structures showed a high correlation to increasing biodiversity in the close environment of the individual plant (Scherling et al., 2010).

As described above, plants show a variety of metabolic responses against varied abiotic stresses. With the availability of genomics platforms, scientists are now able to opt for metabolomics for studying metabolites involving non-biased approaches. However, it would be important to study if there are any common metabolic responses associated with all the abiotic stresses or if the responses are specific to the stress. In this context, Komatsu et al. (2011) identified 81 metabolites related to the mitochondria under flooding stress in roots and hypocotyls of soybean using CE-MS which showed that the TCA-cycle-related metabolites, glycolysis related metabolites, GABA, pyruvate, NAD, NADH and amino acids increased, while ATP decreased. This kind of accumulation/increase in GABA and amino acids was also reported in Lotus unlike TCA cycle related metabolites were found to be low in Lotus and high in soybean. These studies support that integrative analysis is required on the metabolite accumulation within the context of understanding metabolic responses to stress.

Phosphorous is an essential component of energy metabolism, signaling molecules, and structural macromolecules. Therefore, studies have been conducted to understand the role of phosphorus in stress response metabolite profiling of common bean roots and nodules under P starvation (Hernández et al., 2007). Increase in the levels of most of the amino acids and several sugars was reported in P-stressed roots. It was suggested that the accumulation of sugars may be partitioned preferentially to P-stressed roots to support the expression of P stress-induced genes. On the other hand reduced amounts of organic acids are reported in P-starved roots that likely reflect exudation of these metabolites from the roots into the rhizosphere (Hernández et al., 2007). The metabolic response of P-starved nodules is in contrast to that of P-starved roots in common bean. It was reported that amino acids, N-containing metabolites and sugars were decreased, while organic acids were accumulated in P-deficient nodules (Hernández et al., 2009). Such contrasting response may be due to the suppression of N supply from fixed N_2_ under environmental limitations such as P-starvation in nodules. Recently, metabolite profiling study in chickpea revealed 49 primary metabolites in contrasting salt stress responsive cultivars (Dias et al., 2015).

One drawback with the metabolite profiling is that the data obtained is not sufficient to determine the regulation mechanisms of the pathways of interest. In order to overcome this issue, integrated analysis of metabolomics data together with that of proteomic and transcriptomic data need to be performed. For example in Medicago, an integrated metabolomics and transcriptomic approach was found useful to study metabolic reprogramming of the border cells in roots through cumulative and pathway specific datasets (Watson et al., 2015). This integrative approach showed that there were significant differences in the levels of phytohormone, supported by variation in lipoxygenases and auxin responsive transcripts in the border cells and root tips. Additionally, this approach identified metabolic resources for growth and development redirected to the border cells for the accumulation of specialized metabolites that were defense and symbiosis related. Similarly, Komatsu et al. (2011) used an integrative proteomic and metabolomics approach which was useful in identifying the expression and regulation of components linked to flooding stress in soybean seedlings. Larrainzar et al. (2009) integrated GC-TOF-MS metabolite profiling with untargeted and targeted proteomics to reveal nodule metabolic responses under drought stress and recovery by re-watering in Medicago. Metabolite profiling was able to show a highly pronounced reprogramming of metabolism during drought response and the ability of the nodules to recover completely after re-watering.

### Targeted and untargeted metabolite analysis

Metabolites are analyzed either using a targeted or untargeted method (Patti et al., 2012). The targeted method is used to analyse a specified set of metabolites which targets one or more pathways of interest and involves the setup of selected reaction monitoring methods of the standard compounds of the metabolite of interest, followed by the extraction of metabolites from the sample and analysis. The data obtained provides quantitation based on standardized methods for the metabolites of interest. This method has been widely used to follow the dynamics of a limited number of metabolites known to be involved in a particular stress and also for comparative metabolite profiling of a large number of known metabolites. For example, in a single chromatographic run, highly parallel targeted assays based on SRM can be used for sensitive simultaneous analysis of over 100 metabolites (see Bajad and Shulaev, 2007). Alternatively, quantitative profiling may provide *in vivo* enrichment of metabolites with stable isotopes like C-13 and N-15. This can be possible only by growing plants or plant cells in liquid media containing N-15-labeled inorganic nitrogen sources or C-13-labeled carbon dioxide or glucose (Hegeman et al., 2007; Huege et al., 2007). Metabolic labeling combined with MS has been successfully used for quantitative metabolic profiling in microorganisms (Mashego et al., 2004; Lafaye et al., 2005; Wu et al., 2005).

On the other hand, the untargeted metabolite profiling is often used for global and broader applications, e.g., understanding cellular metabolism. In this approach, metabolites are isolated from samples followed by the LC-MS analysis. The data obtained is processed using bioinformatics and values for peaks of interest are searched against metabolite databases for possible identity. These metabolite identities are confirmed using tandem MS (MS/MS) data and retention time data compared with standard components. Untargeted metabolite profiling in Lotus demonstrated a major and reproducible change of the metabolic phenotype in the course of salt acclimatization, which was most evident for amino acid, sugars and organic acid metabolism (Sanchez et al., 2008). Accumulation of amino acids and other nitrogen-containing compounds is a remarkable biochemical feature of almost all plant stress responses reported so far. However, the main disadvantage of untargeted profiling is that it is a semi-quantitative method and provides relative concentration data based on the same “surrogate” internal standard. These semi-quantitative data have to be further validated using targeted quantitative assays.

### Metabolic fingerprinting

Metabolic fingerprinting is mainly used to identify metabolic signatures, for example, finding patterns associated with a particular stress response without precise quantitation/identification of different metabolites in the given sample. Features specific to a fingerprint can be identified using a variety of pattern recognition and multivariate statistical analysis (e.g., principal component analysis (PCA), self-organizing maps (SOMs) and hierarchical clustering, discriminant function analysis (DFA), ANOVA etc.) on the data (Sumner et al., 2003). Different analytical techniques, including NMR spectroscopy (Krishnan et al., 2005), MS (Goodacre et al., 2003), and FT-IR (Johnson et al., 2003) may be used to perform metabolic finger printing. Of these, MS is advantages over NMR spectroscopy because of the low sensitivity of NMR, which makes it difficult to detect low abundance cellular metabolites. On contrary, MS has high resolving power compared to NMR, providing higher sensitivity and lower detection limit but generates more complex spectrum because of its results in the form of discriminant ions which remains as a challenge for data validation. Moreover, a larger subset of metabolites associated with the phenotype can be identified using MS.

There have been few studies that reported the application of these approaches in legumes. Metabolic finger printing has been utilized to study drought in seven different model and forage species of the Lotus genus. Analysis using PCA of the metabolite features regardless metabolite identification status yielded sample “fingerprints,” which classified primarily according to the genotype. Infrared (IR) spectroscopy has been used to obtain a snapshot of the sample metabolome (typically low-molecular-weight compounds) at a given time. This study identified metabolic compounds responsible for rapid fermentation for the efficient conservation of forage proteins (Johnson et al., 2004). In another study, NMR-based approach has been used for metabolic fingerprinting of 21 grass and legume cultivars. Applying PCA, variation between cultivars and the magnitude of changes in the metabolic fingerprint between the spring growth and the second regrowth was elucidated in the study. Furthermore, variation in metabolic compounds such as malic acid, choline, and glucose was reported due to seasonal change (Bertram et al., 2010).

### Metabolomics databases for legumes

As with transcriptomics and proteomics data, metabolomics approaches also generate huge datasets that require specialized data mining and bioinformatics tools. It is imperative to integrate functional genomics data to comprehensively study biological components, using a systemic approach, e.g., through the mathematical modeling of biological systems. Metabolomics data handling, mining and analysis etc. have been improved tremendously, due to advances in bioinformatics tools. In this scenario, several databases have been developed for plant metabolomics data analysis. For example, a metabolic pathway reconstruction was used to generate a pathway database for Medicago called MedicCyc (http://www.noble.org/MedicCyc/) which features more than 250 pathways with related genes, enzymes and metabolites (Urbanczyk-Wochniak and Sumner, 2007). The database contains Medicago specific pathways including isoflavonoid, lignin and triterpene saponin biosynthesis which were added or modified based on literature and in-house expertise. MedicCyc is designed to visualize functional genomics datasets from Medicago within the biological context of metabolic pathways and has been believed that this is best achieved through the visualization of data within the biological context of metabolic pathways in legumes. The pathways were engineered to enable the correlated visualization of integrated functional genomics data. Another database, Soybean Knowledge Base (SoyKB) (http://soykb.org) has been reported to be a comprehensive resource for soybean translational genomics and contains integrative information on soybean genomics, transcriptomics, proteomics and metabolomics (Joshi et al., 2014). This is a web resource that would not only be useful for soybean translational genomics, but also for legume crop improvement programs. Also, a database for plant metabolomics, PlantMetabolomics.org (PM) (http://www.plantmetabolomics.org) was developed (Bais et al., 2010). This database represents metabolomics data generated from Arabidopsis through an integration of experiments compiled from different platforms with visualization tools. PlantMetabolomics has been widely used for exploring, visualizing and downloading plant metabolomics data and well-annotated metabolomics datasets which is useful for establishing metabolomics as a functional genomics tool in legumes.

### Metabolomics for crop breeding

There is growing interest in using metabolites as selection markers in crop breeding programs, because metabolite biomarkers have been linked with strong environmentally-controlled traits (Steinfath et al., 2010). Mapping and metabolomics genome wide association studies (mGWAS) have been conducted to develop “metabotypes” using metabotype quantitative trait locus (mQTL) (association of genomic markers and metabolic markers) which enabled the associations between metabolic concentrations and genetic polymorphisms. Overall, plant metabolomics has benefited from a rich array of pre-existing methodological approaches and bioanalytical knowledge for the characterization of the chemically diverse classes of metabolites. However, speedy progress in the application of these approaches in legumes will be quite useful for legume improvement.

## Summary and future outlook

The development in the area of proteomics and metabolomics has enhanced the power of “omics” with the possibility of studying at different levels of plant regulations, namely transcriptome, proteome and the metabolome (Shulaev et al., 2008). Plant growth, development and stress responses are not straightforward to be able to understand by just looking into one or two level(s), which has been done traditionally. For instance, to understand the molecular basis of stress physiology and biochemistry, the quantitative correlation of different protein groups and classes of metabolites with stress levels are required for the identification of candidate bio-markers (Weckwerth, 2011b; Rodziewicz et al., 2014). Moreover, with the recent developments in the technologies, availability of legume genomics and protein databases, it would be possible to have efficient and high throughput identification of stress related proteins in crop legumes (Hiremath et al., 2011; Kosová et al., 2011; Varshney et al., 2013a; Hossain and Komatsu, 2014a,b).

Legume crops are widely cultivated in the semi-arid tropics where various abiotic and biotic stresses pose severe threats to the productivity. The crops are subjected to not only one stress, but also a combination of stresses at a given point of time under natural field conditions. To sustain legume cultivation under these environments, crop improvement programs requires resourceful methods such as integrated “omics” approaches to understand stress responses at the molecular level such as cellular mechanisms, signaling pathways, biochemical processes. Such studies have largely been initiated in model plants such as Arabidopsis. For instance, transcriptomics and the metabolomics data revealed a different pattern of defense response in Arabidopsis subjected to a combination of drought and heat stress. It was found that proline (Pro) was accumulated in response to drought stress, but during the combination of stresses, Pro was replaced by sucrose as osmoprotectants. Similar studies needs to be undertaken in legume crops such as chickpea, pigeonpea and peanut, which are of agronomic value especially in the semi-arid tropics.

**Figure 1:**
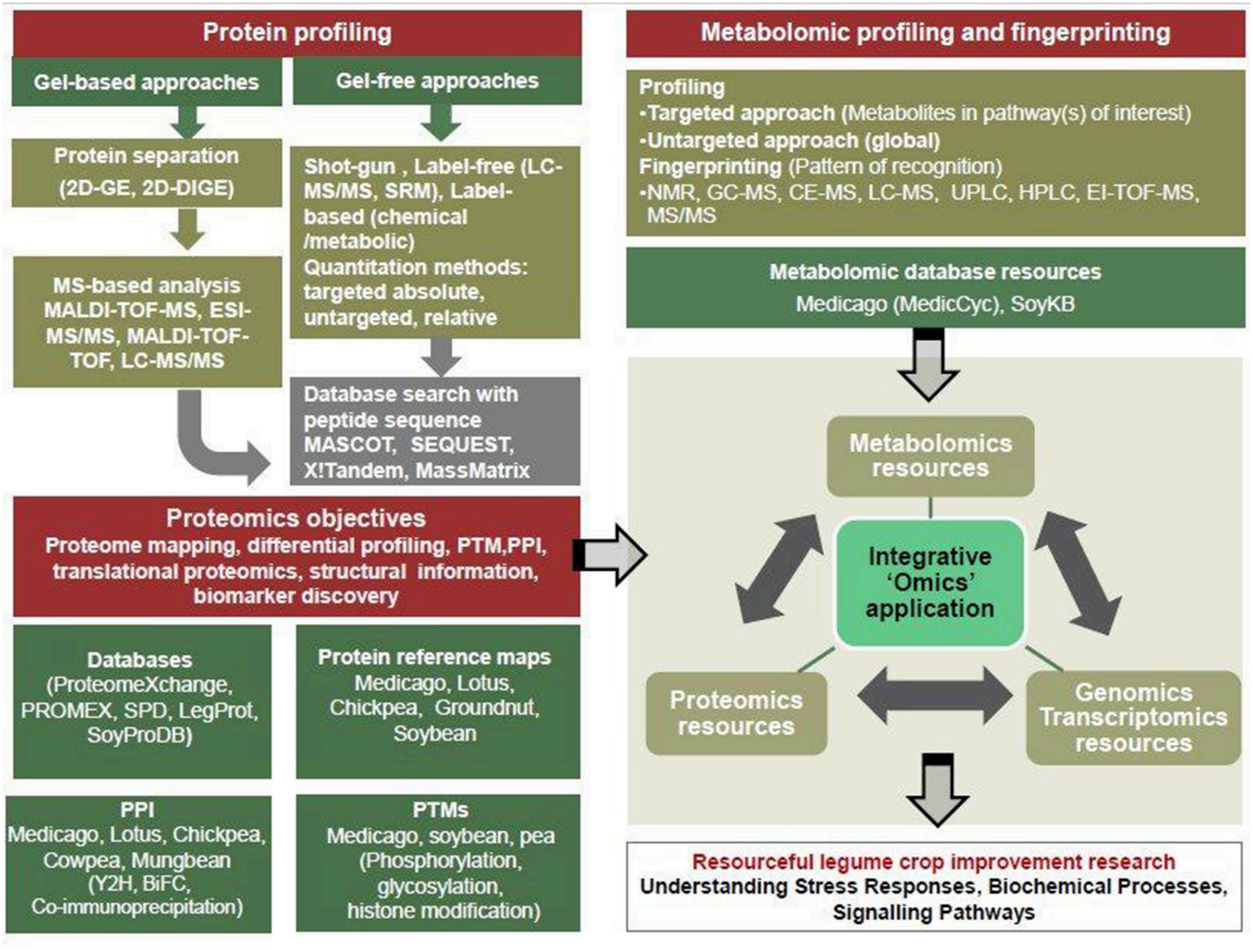
**Significant advances in proteomic and metabolomics for integrative “omics” approaches in legume crop research**.

In this review, the available proteomics and metabolomics resources for legume research and their applications for further our understanding of the stress biology in model as well as crop legumes have been provided (Figure 1). A large number of protein reference maps for various tissues are already available for model legume crops that will greatly support comparative proteomics approaches. There is a great need to generate proteome maps from crop legumes that would greatly help in precise understanding of various cellular processes and signaling pathways. These resources would improve the functional annotations of the gene models in identifying novel ORFs as in the case of Arabidopsis (Castellana et al., 2008) and also validates the existing annotation (Agrawal et al., 2012, 2013; Dash et al., 2015; Walley and Briggs, 2015). Moreover, it is now possible to uncover the regulation of these processes by studying the post-translational modifications. Further, huge amount of information on metabolomics data related to stress responses in legumes have been generated which includes a large number of metabolic pathways under stress conditions. The integrative analyses of data through genomics, transcriptomics, proteomics and metabolomics will be important for a system biology approach and for efficient application in legume crop improvement. At this point, it would be worthwhile to caution that combining and integrating omics data together is challenging, because of the difficulty in correlating the data due to the differences in the time for quenching metabolism. In addition, some errors can be introduced due to the nature of the samples used and the lack of appropriate mathematical models that would allow identification of various biochemical and signaling pathways involved in stress response. Therefore, there is a need to advance at all the fronts, i.e., sampling strategy, data generation, data analysis, etc. in different disciplines so that precise system biology approach can be deployed to understand molecular basis of different traits related to plant biology as well as breeding applications.

### Conflict of interest statement

The authors declare that the research was conducted in the absence of any commercial or financial relationships that could be construed as a potential conflict of interest.
